# The Medical Society of the County of Erie

**Published:** 1909-04

**Authors:** Franklin C. Gram

**Affiliations:** Secretary


### SOCIETY PROCEEDINGS.

### The Medical Society of the County of Erie.

[Carbon copy supplied February 19, 1909.]

By FRANKLIN C. GRAM, M. D., Secretary.

THE regular meeting of the Medical Society of the County of Erie was held Monday evening, February 15, 1909, in the rooms of the Society of Natural Sciences, Buffalo Library Building.

The president Dr. Charles A. Wall called the meeting to order at 8.30 o'clock, and requested the secretary to read the minutes of the annual meeting held December 21, 1908. On motion, the minutes were adopted without reading.

The secretary then read the minutes of the council meeting held February 6, 1909. On motion of Dr. Krauss, the minutes of the council meeting were approved and the recommendations contained therein adopted.

This included the recommendation that honorary membership be conferred upon Dr. Henry R. Hopkins, who is thereby made an honorary member of the society; also the resignations of Drs. W. H. Glenny and Dewitt G. Wilcox, Buffalo, Henry Y. Grant, Falls View, Ont., A. G. Gumaer, Los Angeles, Cal., and A. P. Squires. Resignations of these members were thereby accepted.

The secretary stated that in response to a request sent to State Secretary Townsend, at the direction of the council, for a copy of report on malpractice suits defended by the State Society during 1908, he had received a complete report made by the counsel for the State Society.

A summary of this report was then read. The entire report will be published in the journal of the State Society.

The president called upon Dr. Eli H. Long, secretary of the state delegation, to make a report of the last meeting of the State Society. Dr. Long presented a verbal report of the work done by the delegates from this county and the results accomplished.

Dr. Lytle offered the following amendment to the by-laws: "That Section 8 of Chapter II. be amended by substituting the words 'is dropped from the roll for non-payment of dues' in place of the words 'removes from the State of New York permanently.'"

Under the rules, this amendment was laid upon the table, a copy to be sent to each member, the same to be acted upon at the next meeting.

Dr. Allen A. Jones suggested the advisability of employing a stenographer to make a verbatim report of the discussions at these meetings to be published in the State Society's Journal. The matter was referred to the council with power.

Dr. William C. Krauss nominated Dr. Ernest Wende for honorary membership. This, under the rules, was referred to the council.

On the recommendation of the Membership Committee, the following persons, whose names were presented by chairman Thomas H. McKee, were elected to membership in this society: Edward H. Kramer, 64 North Ogden street, Buffalo; W. B. Burlingham, 260 W. Utica street, Buffalo; Aldona Janowski, 472 Amherst street, Buffalo; Geo. J. Haller, 1084 Main street, Buffalo; Irving Phillips Lyon, 531 Franklin street, Buffalo.

The business portion of the session now being completed, the scientific program of the evening was proceeded with. L. Bradley Dorr presented a paper on "Silver Salts in Typhoid Fever;" John H. Grant spoke on "Whitlow or Felon;" F. C. Busch made brief remarks on "Practical Application of Research;" Jacob S. Otto spoke on "Tendencies in Infant Feeding;" Joseph Burke presented the subject, "Diagnosis of Pancreatic Affections," (published elsewhere in this number). These papers were discussed, a number of the members participating.

At the conclusion of the program a collation was served. About seventy-five members and guests were present.

